# Pregnancy outcomes among patients with complex congenital heart disease

**DOI:** 10.1038/s44325-024-00022-w

**Published:** 2024-09-17

**Authors:** Jiaqi Gu, He Zhao, Jun Zhang

**Affiliations:** Department of Obstetrics and Gynecology, Beijing Anzhen Hospital, Capital Medical University, Beijing, China

**Keywords:** Congenital heart defects, Heart development

## Abstract

Patients with complex congenital heart disease (CCHD) may pose a serious threat to the mother-infant safety. This study intends to explore the influencing factors for adverse pregnancy outcomes in the CCHD population. Totally 108 CCHD patients who terminated pregnancy from January 2013 to January 2023 were recruited. We collected clinical data during the pregnancy from electronic medical records. Among them, 45 patients had adverse pregnancy outcomes (41.7%) and no patient died. 5 patients with no newborn. The incidence rate of adverse pregnancy outcomes was significantly higher in patients with brain natriuretic peptide (BNP) > 100 pg/mL (OR: 2.736; 95%CI: 1.001–7.481, *p* = 0.049) and hypoxemia (OR: 15.46; 95%CI: 1.689–141.512, *p* = 0.015) and without undergoing cardiac surgical correction (OR: 3.226; 95%CI: 1.121–9.259, *p* = 0.03). It was confirmed by propensity score matching that no cardiac surgical correction was an independent risk factor. Maternal patients without undergoing cardiac surgical correction had poorer NYHA cardiac function (*p* = 0.000) and were more prone to heart failure (*p* = 0.027), hypoxemia (*p* = 0.042), pulmonary arterial hypertension (*p* = 0.038) and postpartum hemorrhage (*p* = 0.016). Moreover, these patients had prolonged Surgical Intensive Care Unit (SICU) stay (*p* = 0.000) and significantly higher risk of premature delivery (*p* = 0.005), low birth weight (*p* = 0.018), infection and asphyxia (*p* = 0.043). Corrective cardiac surgery in patients with CCHD before pregnancy significantly reduces the incidence of adverse pregnancy outcomes.

## Introduction

The number of pregnancies in women with complex congenital heart disease (CCHD) has increased over the past decades and is expected to rise further in the coming years^1^. The severe abnormalities of cardiac structure and hemodynamic changes during pregnancy of CCHD patients bring greater challenges to the cardiovascular system^2^. Due to the increases in the maternal blood volume, cardiac output, oxygen consumption and heart rate during pregnancy, the patients may develop heart failure, malignant arrhythmia and other serious complications at any time, leading to high mortality and extremely high risk of perinatal adverse pregnancy outcomes^3^. According to a study by the European Registry of Pregnancy and Cardiac Disease (ROPAC), only 40% of CCHD patients have no adverse pregnancy outcomes, and maternal events and fetal events occur in about 36% and 43% of them, respectively^4^. Recent studies have shown that brain natriuretic peptide (BNP), low oxygen saturation, pulmonary arterial hypertension and other risk factors can significantly affect the pregnancy outcome of the CCHD population^5,6^. At present, the pre-pregnancy assessment, pregnancy management and reduction of the incidence of adverse pregnancy outcomes in this population are one of the focuses of attention.

Currently, numerous advancement have been made for the patient suffering from CCHD during pregnancy, which significantly improve the outcomes^7^. In particular, CCHD surgical techniques have improved^8^, but there is still a lack of evidence whether surgery can improve the prognosis of pregnant women with CCHD. This study intends to explore the influencing factors for adverse pregnancy outcomes in the CCHD population, so as to further improve the clinical management and intervention of the disease, and provide guidance for obtaining favorable pregnancy outcomes.

## Methods

### Patients

This study includes pregnant women with CCHD who were hospitalized in Beijing Anzhen Hospital from January 2013 to January 2023. In 2018, the American Heart Association proposed the AP classification of congenital heart disease, i.e., Anatomy + Physiological stage, to assess the complexity of congenital heart disease, in which all IA-IIID grades can be classified as CCHD: including types such as Tetralogy of Fallot, corrected transposition of the great arteries, complete transposition of the great arteries, Ebstein’s anomaly, single ventricle, and severe valvular anomalies^9^.

Inclusion criteria were as follows: (1) Pregnant women with CCHD; (2) those who terminated pregnancy at <28 weeks for cardiac reasons and for all reasons at ≥28 weeks. Exclusion criteria were as follows: (1) Patients with incomplete data; (2) those who terminated pregnancy for non-cardiac reasons at <28 weeks. A total of 108 eligible patients were included.

The study protocol was approved by the ethics committees or institutional review boards of the Beijing Anzhen Hospital. All clinical procedures fulfilled the tenets of the Declaration of Helsinki. All participants were informed about the study’s purpose, risks, and benefits and gave informed consent for this study. Supplementary Fig. 1 shows the flowchart of the current study scheme.

### Prognostic variables

The data related to pregnancy were mainly collected prior to delivery, including maternal CCHD type, age, parity, length of SICU stay, NYHA cardiac function class, mode of delivery, history of cardiac surgery, postpartum hemorrhage, arrhythmia, valvular regurgitation, Eisenmenger, heart failure, neonatal Apgar score, birth weight, gestational age at birth, neonatal malformation, neonatal complications (neonatal infection, asphyxia, hyperbilirubinemia, arrhythmia), echocardiographic indicators (ejection fraction, pulmonary arterial pressure, valvular regurgitation), BNP, hemoglobin, and SPO_2_.

### Adverse pregnancy outcomes

The adverse pregnancy outcomes include adverse maternal outcomes and adverse neonatal outcomes. Adverse maternal outcomes were defined as having any of the following conditions. Termination of pregnancy was characterized by a gestational age of delivery <37 weeks. Postpartum hemorrhage was defined as blood loss of more than 500 ml after vaginal delivery or more than 1000 ml after cesarean section^10^. Miscarriage was identified as the spontaneous loss of pregnancy before 28 weeks of gestation. Arrhythmia was characterized by irregular heartbeats detected during pregnancy through electrocardiogram (ECG). Admission to the Surgical Intensive Care Unit (SICU) was necessitated by severe complications such as severe infection^11^. Heart failure was defined as a cardiac ejection fraction less than 50% accompanied by symptoms such as dyspnea^12^. Adverse neonatal outcomes included conditions such as low birth weight, defined as a birth weight of ≤2500 grams. Neonatal infection was diagnosed based on clinical signs and laboratory findings indicative of infection, such as fever, lethargy, and positive blood cultures. Neonatal asphyxia was defined by an Apgar score of less than 8 at 5 min, indicating difficulty in establishing adequate breathing at birth. Neonatal malformations were congenital abnormalities detected in the newborn through clinical examination or imaging studies. Any combination or single occurrence of the above outcomes in the same patient was counted as one adverse pregnancy outcome.

### Statistical analysis

SPSS25.0 software was used for data analysis. Measurement data were compared between the two groups by *t*-test. Enumeration data were expressed as [n(%)], and compared between the two groups by chi-square test or Fisher’s exact probability test. Multivariate Logistic regression analysis was performed on statistically significant variables in univariate analysis (BNP > 100 pg/mL, without a history of cardiac surgery, cardiac function, pulmonary arterial pressure, and hypoxemia). The propensity score matching method was used to reduced selection bias introduced by using a non-randomized design. The following variables were used to compute the propensity score for each patient: age, BNP, Hypoxemia, NYHA Cardiac function class, Arrhythmia, Hemoglobin, ejection fraction and PAP. *p* < 0.05 was considered statistically significant. Using Graphpad Prism for Statistical Mapping. R software was used for data visualization.

The propensity score matching method was used to reduced selection bias introduced by using a non-randomized design. The following variables were used to compute the propensity score for each patient: age, BNP, hypoxemia, NYHA Cardiac function class, arrhythmia, hemoglobin, ejection fraction and PAP. Confounding bias due to unknown factors cannot be completely excluded with this study design.

## Results

### The general characteristics of the study population

In this study, the average age of the 108 CCHD patients was 29 ± 4 years. Most patients had good cardiac function (87%). Among the pregnant women, 31.5% had elevated BNP levels (>100), indicating potential cardiac stress. The mode of delivery was predominantly cesarean section (86.4%). Baseline information on the 108 pregnant women with complex pre-centers is shown in Table 1.

**Table 1:** Baseline information on pregnant women with CCHD

Stage of patients	Characteristic	*N* = 108
CCHD pregnants before delivery	Age (years)	29 ± 4
	BNP > 100, *n* (%)	34 (31.5)
	PAP	
	Mild, *n* (%)	18 (16.7)
	Moderate, *n* (%)	12 (11.1)
	Severe, *n* (%)	2 (1.9)
	NYHA Cardiac function class ≥III, *n* (%)	14 (13)
	Hemoglobin (g/L)	124 ± 17.9
	Oxygen saturation <90%, *n* (%)	12 (11.1)
	A history of cardiac surgery, *n* (%)	75 (69.4)
	Preeclampsia, *n* (%)	8 (7.4)
	Eisenmenger, *n* (%)	1 (0.9)
	Visceral inversion, *n* (%)	5 (4.6)
CCHD pregnants after delivery	CS, *n* (%)	89 (86.4)

*BNP* Brain Natriuretic Peptide, *NYHA* Brain Natriuretic Peptide, *SICU* surgery intensive care unit, *PAP* Pulmonary arterial hypertension, *CS* cesarean section.

### The adverse pregnancy outcome

The average gestational age at termination of pregnancy was 36 ± 4 weeks. There were 5 cases of miscarriage (4.63%), with no maternal mortality events reported. Their primary mode of delivery was cesarean section (86.4%), with a few newborns presenting complications (6.81%). Maternal outcomes in Table 2 and neonatal outcomes in Table 3.

**Table 2:** Maternal outcomes in adverse pregnancy outcomes

Stage of patients	Characteristic	*N* = 108
maternal	Termination of pregnancy (weeks)	36 ± 4
	Postpartum hemorrhage, *n* (%)	11 (10.7)
	Miscarriage, *n* (%)	5 (4.6)
	Arrhythmia, *n* (%)	21 (19.4)
	Admission to SICU, *n* (%)	34 (31.5)
	Heart failure, *n* (%)	3 (2.8)

**Table 3:** Neonatal outcomes in adverse pregnancy outcomes

Stage of patients	Characteristic	*N* = 103
neonatal	Birth weight ≤2500 g, *n* (%)	26 (25.2)
	Neonatal infection, *n* (%)	4 (3.9)
	Neonatal asphyxia, *n* (%)	3 (2.91)
	Neonatal malformation, *n* (%)	7 (6.8)

### The adverse outcomes in different types of CCHD

The types of CCHD included tetralogy of Fallot, congenitally corrected transposition of the great arteries, complete transposition of the great arteries, Ebstein’s anomaly, single ventricle, double outlet of the right ventricle, aortic stenosis, and pulmonary stenosis (Table 4). Among them, tetralogy of Fallot accounts for the majority (67.59%), followed by transposition of the great arteries (18.52%). Complete transposition of the great arteries and double outlet of the right ventricle have the highest incidence of adverse pregnancy outcomes (100%).

**Table 4:** Incidence of adverse pregnancy outcomes in patients with different types of CCHD

CCHD	Residual cardiac disease	*N* (%)	Adverse pregnancy outcome	Percentage(%)
TOF	-	62(57.41)	22	36.99
	DAA	1(0.93)	1	
	PS	3(2.78)	0	
	RAA	1(0.93)	0	
	ASD	2(1.85)	1	
	VSD	2(1.85)	1	
	PAVM	1(0.93)	1	
	PLSVC	1(0.93)	1	
ccTGA	-	8(7.41)	3	41.18
	VSD,PS	1(0.93)	0	
	EA	1(0.93)	0	
	ASD,VSD,PS	3(2.78)	1	
	VSD	3(2.78)	2	
	AVL,AS	1(0.93)	1	
TGA	SA,SV,PS	1(0.93)	1	100
	SV,ASD	2(1.85)	2	
EA	-	4(3.7)	2	57.14
	ASD	3(2.78)	2	
DORV	VSD,PS	1(0.93)	1	100
	VSD,MVP	1(0.93)	1	
	ASD,VSD,PS,MA	1(0.93)	1	
AS	AVL	1(0.93)	0	0
PS	VSD,AMBRV	1(0.93)	1	33.33
	RVOTS	2(1.85)	0	
CTA	ASD	1(0.93)	0	0

*ADR* Atrial defect repair, *AMBRV* Anomalous muscle band of right ventricle, *AS* Aortic stenosis, *ASD* Atrial septal defect, *AVL* Aortic valve leafletization, *BGP* Bidirectional Glenn procedure, *BPV* Balloon Pulmonary Valvuloplasty, *CCHD* Complex congenital heart disease, *ccTGA* Congenitally corrected transposition of the great arteries, *CTA* Cor triatriatum, *DAA* Double aortic arch, *DORV* Double outlet of the right ventricle, *EA* Ebstein’s anomaly, *MA* Mitral atresiam, *MVP* Mitral valve prolapse, *MVR* Mitral valve replacement, *PAC* Pulmonary artery circumflexion, *PAVM* Pulmonary arteriovenous malformation, *PFO* Patent foramen ovale suture, *PI* Pacemaker insertion, *PLSVC* Permanent left superior vena cava, *PR* Patch repair, *PS* Pulmonary stenosis, *PV* Pulmonary valvotomy, *RVOT* Right ventricular outflow tract unblocking, *RVOTS* Right ventricular outflow tract stenosis, *SA* Single atrium, *SV* Single ventricle, *TCPC* Total cavopulmonary connection, *TGA* Transposition of the great arteries, *TOFRS* Tetralogy of Fallot radical surgery, *TPVR* Transcatheter pulmonary valve replacement, *TV* Tricuspid valvuloplasty, *VIB* Ventricular isthmus blockage, *VSD* Ventricular septal defect.

### The adverse outcomes in different patients with or without surgery

We analyzed the adverse outcomes in different patients with or without surgery. We found that the incidence of adverse outcomes in patients after cardiac surgery (32.88%) was significantly lower than that in patients without surgery (60%) (*p* < 0.001). Detailed results are shown in Table 5.

**Table 5:** Cardiac surgical status and incidence of adverse pregnancy outcomes in patients with CCHD

Cardiac Surgery Status	CCHD correction (simultaneous surgery)	Secondary correction (other cardiac surgeries)	*N* (%)	Adverse preginterventionnancy outcome(%)
With cardiac surgery			73(67.6%)	24 (32.9)
	TOFRS	-	55(50.9)	16
	TOFRS, VIB	-	1(0.93)	0
	TOFRS, ADR	-	1(0.93)	0
	TOFRS, PFO suture	-	1(0.93)	1
	TOFRS	VIB	1(0.93)	0
	TOFRS	PR	1(0.93)	0
	TOFRS	PS	1(0.93)	1
	TOFRS	TPVR	1(0.93)	0
	TOFRS	BPD	1(0.93)	0
	ADR,VIB,PV	PI	1(0.93)	0
	BGP	-	4(3.7)	4
	BGP	PAC	1(0.93)	1
	TCPC	-	1(0.93)	1
	RVOT	-	1(0.93)	0
	Type of surgery not available	-	2(1.85)	0
Without cardiac surgery			35(32.4)	21(60%)
	Uncorrected	MVR,TV	1(0.93)	0
	Uncorrected	-	32 (29.63)	19
	No chance of surgery	-	2(1.85)	2

*ADR* Atrial defect repair, *BGP* Bidirectional Glenn procedure, *BPV* Balloon Pulmonary Valvuloplasty, *CCHD* Complex congenital heart disease, *MVR* Mitral valve replacement, *PAC* Pulmonary artery circumflexion, *PI* Pacemaker insertion, *PFO* Patent foramen ovale suture, *PR* Patch repair, *PS* Pulmonary stenting, *PV* Pulmonary valvotomy, *RVOT* Right ventricular outflow tract unblocking, *TCPC* Total cavopulmonary connection, *TOFRS* Tetralogy of Fallot radical surgery, *TPVR* Transcatheter pulmonary valve replacement, *TV* Tricuspid valvuloplasty, *VIB* Ventricular isthmus blockage.

### Factors associated with adverse outcomes

According to the presence or absence of adverse pregnancy outcomes, the pregnant patients with CCHD were divided into two groups. The incidence rate of adverse pregnancy outcomes was significantly higher in patients with BNP > 100 pg/mL (*p* = 0.014), hypoxemia (*p* = 0.001), NYHA cardiac function class ≥III (*p* = 0.001) and elevated pulmonary arterial pressure (*p* = 0.007), and without undergoing cardiac surgical correction (*p* = 0.004) in both groups (Table 6). There were no statistically significant differences in age, arrhythmia, gravidity and parity, Hb and EF between the two groups (*p* > 0.05).

**Table 6:** Univariate analysis of adverse pregnancy outcomes in pregnant patients with CCHD

Influencing factor	No adverse pregnancy outcome (*n* = 63)	Adverse pregnancy outcome (*n* = 45)	*P* Value
BNP > 100, *n* (%)	14 (22.22)	20 (44.44)	0.014
Hypoxemia, *n* (%)	1 (1.589)	11 (24.44)	0.001
NYHA Cardiac function class ≥III, *n* (%)	2 (3.17)	12 (26.67)	0.001
A history of cardiac surgery, *n* (%)	50 (79.37)	24 (53.33)	0.004
Arrhythmia, *n* (%)	14 (22.22)	7 (15.56)	0.388
Age (years)	29 ± 4	28 ± 5	0.692
hemoglobin (g/L)	121.22 ± 14.713	127.96 ± 21.099	0.053
ejection fraction (%)	61.10 ± 5.713	60.62 ± 6.645	0.693
PAP (mmHg)	25.214 ± 11.249	33.789 ± 18.528	0.007

*CCHD* Complex congenital heart disease, *BNP* Brain Natriuretic Peptide, *NYHA* Brain Natriuretic Peptide, *PAP* Pulmonary arterial hypertension.

**Fig. 1:**
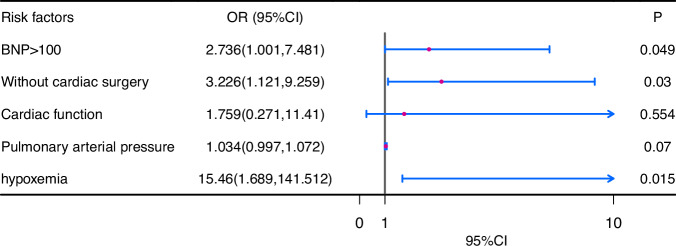
Analysis of risk factors for adverse pregnancy outcomes. Legend: Logistic regression analysis was performed on statistically significant variables identified in the univariate analysis, including BNP levels greater than 100 pg/mL, absence of a history of cardiac surgery, cardiac function, pulmonary arterial pressure, and hypoxemia.

The results of univariate analysis showed that BNP > 100 pg/mL, no cardiac surgical correction, NYHA cardiac function class ≥III, elevated pulmonary arterial pressure and hypoxemia were all risk factors for adverse pregnancy outcomes (*p* < 0.05). Then they were incorporated into the multivariate logistic regression analysis. It was found that BNP > 100 pg/mL (OR: 2.736; 95%CI: 1.001–7.481, *p* = 0.049), no cardiac surgical correction (OR: 3.226; 95%CI: 1.121–9.259, *p* = 0.03) and hypoxemia (OR: 15.46; 95%CI: 1.689–141.512, *p* = 0.015) were independent risk factors for adverse pregnancy outcomes (Fig. 1).

### Association of cardiac surgical correction and adverse pregnancy outcomes

In order to make the baseline information consistent between the two groups with and without cardiac surgical correction, we chose the propensity score matching method. This approach was used to further investigate the independent impact of preoperative refinement of cardiac corrective surgery on adverse pregnancy outcomes in patients with complicated congenital heart disease.

In the unmatched data, there were 75 individuals in the group who underwent cardioversion before pregnancy and 33 individuals in the group who did not. After propensity score matching, we obtained 33 pairs of matched data. One-way analyses of the matched variables were conducted again, showing no statistically significant differences in the remaining baseline information, confirming that the match was established (Table 7).

**Table 7:** Baseline characteristics of unmatched and propensity matched patients

	Unmatched			Propensity Matched		
	No cardiac surgical correction (*n* = 33)	Cardiac surgical correction (*n* = 75)	*P* Value	No cardiac surgical correction (*n* = 33)	Cardiac surgical correction (*n* = 33)	*P* Value
Age (years)	29.27 ± 5.01	28.40 ± 3.79	0.322	29.27 ± 5.01	27.85 ± 3.61	0.19
PAP(mmHg)	34.47 ± 20.50	26.28 ± 11.56	0.038	34.47 ± 20.50	30.21 ± 12.57	0.31
Hemoglobin(g/L)	131.21 ± 22.08	120.87 ± 14.771	0.018	131.21 ± 22.08	122.82 ± 12.99	0.064
SPO_2_(%)	94.62 ± 5.99	97.02 ± 4.12	0.042	94.62 ± 5.99	95.68 ± 5.87	0.47
ejection fraction(%)	60.27 ± 8.60	61.17 ± 4.63	0.574	60.27 ± 8.60	61.64 ± 4.60	0.42
NYHA Cardiac function class ≥III, *n* (%)	11 (33.33)	3 (4.00)	0.000	9 (27.27)	4 (12.12)	0.138
BNP > 100, *n* (%)	10 (30.30)	24 (32.00)	0.861	10 (30.30)	7 (21.21)	0.398
Arrhythmia, *n* (%)	4 (12.12)	17 (22.67)	0.202	4 (12.12)	6 (18.18)	0.731

*BNP* Brain Natriuretic Peptide, *CCHD* Complex congenital heart disease, *CS* Cesarean section, *NYHA* New York Heart Association, *PAP* Pulmonary arterial pressure, *SICU* Surgery intensive care unit.

We performed a univariate analysis of all matched patients (66 in total) grouped according to whether or not an adverse pregnancy outcome occurred, and the results showed that whether or not cardiac corrective surgery was performed had a significant and independent effect on pregnancy outcome (*p* = 0.001) (Table 8).

**Table 8:** Analysis of factors influencing adverse pregnancy outcomes after matching

Influencing factor	No adverse pregnancy outcome (*n* = 30)	Adverse pregnancy outcome (*n* = 36)	*P* Value
BNP > 100 pg/ml, *n* (%)	3 (10.0)	14 (38.89)	0.839
Arrhythmia, *n* (%)	4 (13.33)	6 (16.67)	0.975
NYHA Cardiac function class ≥III, *n* (%)	1 (3.33)	12 (33.33)	0.006
A history of cardiac surgery, *n* (%)	22 (73.33)	11 (30.56)	0.001
SPO_2_(%)	97.53 ± 2.04	93.17 ± 7.25	0.001
Age (years)	28.43 ± 3.31	28.67 ± 5.17	0.825
hemoglobin (g/L)	122.63 ± 10.83	130.67 ± 22.49	0.064
ejection fraction (%)	60.27 ± 7.14	61.53 ± 6.70	0.463
PAP (mmHg)	28.17 ± 13.07	35.82 ± 19.19	0.068

*CCHD* Complex congenital heart disease, *BNP* Brain Natriuretic Peptide, *NYHA* Brain Natriuretic Peptide, *PAP* Pulmonary arterial hypertension.

Basic data of pregnant patients with CCHD were compared with and without cardiac surgical correction. The results revealed that there were statistically significant differences between the two groups in gestational age at termination of pregnancy (*p* = 0.005), length of SICU stay (*p* = 0.000), pulmonary arterial pressure (*p* = 0.038), Hb (*p* = 0.018), SPO_2_ (*p* = 0.042), cardiac function (*p* = 0.000), incidence of heart failure (*p* = 0.027), and postpartum hemorrhage (*p* = 0.016). Patients without undergoing cardiac surgical correction had significantly higher rate of premature delivery, pulmonary arterial pressure, Hb and incidence of heart failure, significantly longer length of SICU stay, significantly lower SPO_2_, and significantly poorer cardiac function than those undergoing cardiac surgical correction. The delivery mode had no statistically significant difference between the two groups (*p* > 0.05), mainly cesarean section (CS) rather than vaginal delivery (VD). There were 27 cases (93.1%) and 63 cases (85.1%) of CS among patients without undergoing cardiac surgical correction and undergoing cardiac surgical correction, respectively (Table 9). No statistically significant differences were found between the two groups in age, parity, EF, BNP, and incidence of Eisenmenger syndrome and arrhythmia (*p* > 0.05). Taken together, these data suggest that not having cardiac surgery before pregnancy is a risk factor, which also correspond to the odds ratio (3.226) as shown in Fig. 1.

**Table 9:** Comparisons of obstetric complications in CCHD patients with or without undergoing cardiac surgical correction

Indicator	No cardiac surgical correction (*n* = 33)	Cardiac surgical correction (*n* = 75)	*P* Value
Gestational age at termination of pregnancy(weeks)	33.91 ± 6.20	37.24 ± 2.29	0.005
Length of SICU stay(days)	1.12 ± 1.193	0.23 ± 0.628	0.000
Heart failure, *n* (%)	3 (9.09)	0 (0.00)	0.027
Eisenmenger, *n* (%)	1 (3.03)	0 (0.00)	0.306
CS, *n* (%)	27 (93.10)	63 (85.13)	0.444
Postpartum hemorrhage, *n* (%)	7 (24.13)	4 (5.405)	0.016

*CCHD* Complex congenital heart disease, *BNP* Brain Natriuretic Peptide, *NYHA* Brain Natriuretic Peptide, *SICU* surgery intensive care unit, *CS* cesarean section.

There were 103 live births in the two groups. The two groups had significant differences in the birth weight (*p* = 0.018), and incidence of neonatal complications (infection, asphyxia) (*p* = 0.043) (Table 10). Specifically, the incidence of low birth weight, asphyxia, infection and postpartum hemorrhage in patients without undergoing cardiac surgical correction was significantly higher than that in patients undergoing cardiac surgical correction.

**Table 10:** Comparisons of neonatal outcomes in patients with or without undergoing cardiac surgical correction

Indicator	No cardiac surgical correction (*n* = 29)	Cardiac surgical correction (*n* = 74)	*P* Value
Birth weight <2500 g, *n* (%)	12 (41.37)	14 (18.91)	0.018
Neonatal complications (infection, asphyxia), *n* (%)	12 (41.37)	16 (21.62)	0.043

Among the 103 neonates, there were 4 (3.9%) neonatal infections, 3 (2.9%) neonatal asphyxia, 1 (1.0%) neonatal cardiac arrhythmia, and 26 neonates with a birth weight of <2500 g, including 2 very-low-birth-weight infants (1230 g and 1160 g) and 1 very-low-birth-weight infant (980 g), all born prematurely at 30 weeks. The mothers of these three children had one case of heart failure and two cases of concomitant severe pulmonary hypertension.

## Discussion

Pregnancy is a major life event for almost every woman. However, for women with CCHD pregnancy is associated with additional risks and deserves special attention^13^. This study evaluates the possible influencing factors for pregnancy outcomes of patients with CCHD, and the high-risk factors for adverse pregnancy outcomes were searched to predict the occurrence of adverse pregnancy outcomes to a certain extent. After analyzing 108 CCHD patients, we found that BNP, history of cardiac surgical correction, NYHA cardiac function class, pulmonary arterial pressure, and hypoxemia were associated with adverse events. After multivariate adjustment, BNP, history of cardiac surgical correction, and hypoxemia remained associated with adverse events. Patients who did not undergo cardiac surgery had a higher incidence of adverse pregnancy outcomes. Propensity score matching analysis further confirmed the independent effect of cardiac surgery on improving pregnancy outcomes. Patients who did not undergo cardiac surgery had significantly higher rates of preterm birth, pulmonary arterial pressure, heart failure, postpartum hemorrhage, and longer SICU stays. Additionally, these patients had significantly increased rates of low birth weight, asphyxia, and infection in newborns compared to the surgery group.

CCHD is a progressive congenital disease, and the opportunity for surgery may be eventually lost. Patients with CCHD have far inferior cardiovascular adaptability to the population with normal cardiac function, and are faced with serious threats brought about by the unique hemodynamic changes during pregnancy. A German study involving 4015 CHD patients found that, compared to a healthy control group, the incidence of stroke, heart failure, and arrhythmia during pregnancy was significantly higher. As the complexity of CHD increased, the incidence of adverse maternal and fetal outcomes also rose. However, the risk factors remain unclear^14^. Previous studies have shown that high-risk factors for adverse cardiovascular events in patients with congenital heart disease (CHD) include NYHA Class III-IV heart function and heart failure^15,16^. Several studies have shown that a high BNP concentration (>100 pg/mL) is associated with adverse cardiovascular events and adverse maternal-infant outcomes, and BNP currently serves as a negative predictor of adverse events, consistent with the findings in this study^17^. Our study has reached similar conclusions.

Shuenn-Nan et al. argued that the incidence of pulmonary arterial hypertension is up to 49.9% in the CCHD population by the age of 40 years, and Asian CCHD patients with pulmonary arterial hypertension have a higher incidence of adverse events than those without pulmonary arterial hypertension^5^ In this study, the pulmonary arterial pressure in patients who had undergone cardiac surgical correction before pregnancy was significantly lower than that in patients without undergoing cardiac surgical correction. Therefore, cardiac surgical correction before pregnancy in patients with CCHD is beneficial to delaying the progression of pulmonary arterial hypertension, but has less significant effect on adverse pregnancy outcomes. Considering the small sample size, the significance of cardiac surgical correction remains to be determined by more prospective studies.

Among the 108 patients in this study, the incidence of adverse maternal and neonatal outcomes significantly decreased among those who underwent cardiac surgery. Wang et al.^18^ found that cardiac surgical correction before pregnancy in CCHD patients can effectively relieve cardiac dysfunction, improve oxygen saturation, and reduce the incidence of perinatal complications, achieving better pregnancy outcomes in most patients, consistent with the results of this study. However, patients are still at risk at any time after surgery, and adequate risk assessment is required before pregnancy, regardless of the presence or absence of residual cardiac disease^18^.

Patients with CHD often require multiple surgeries, and the timing of surgical intervention should be determined within the context of each patient’s life cycle. Since repeat cardiac surgery is high-risk, the decision to perform surgery must be made with caution, especially in young women of childbearing age. Surgery is usually not performed if residual disease is mild or if pregnancy is tolerable.

For patients with CCHD, surgery usually cannot completely resolve the problem. Although surgery can significantly improve heart structure and function, residual cardiac lesions and long-term complications may still persist. Hemodynamic changes during pregnancy can exacerbate the condition of patients with CCHD. Among these patients, there are currently three common postoperative statuses: post-radical tetralogy of Fallot (TOF), post-arterial switch transposition of the great arteries (TGA), and functional univentricularity after Fontan. For post-radical TOF, long-term complications mainly include residual pulmonary regurgitation, right ventricular dilatation, ventricular dysfunction, and cardiac arrhythmia. Pregnancy may exacerbate these complications but is generally well-tolerated by most patients^19^. For post-arterial switch TGA, long-term complications include anatomical stenosis of the reconstructed great vessels and coronary dysfunction. Pregnancy may lead to neoaortic root dilatation, aortic regurgitation, and myocardial ischemia of coronary origin. Studies have shown that pregnancy is better tolerated in postoperative women. The prognosis for mother and child is extremely poor in those with severely reduced pulmonary blood flow or combined with severe pulmonary vascular disease^20^. In the case of functional univentricularity after Fontan, pregnancy is not recommended for patients with ventricular dysplasia, cyanosis, or mitral valve closure insufficiency. The prognosis for mothers and infants is poorer in patients with severely diminished pulmonary blood flow or comorbid severe pulmonary vascular disease^21^. Our study confirms that the incidence of adverse events is lower in patients with CCHD who have undergone cardiac surgery. Pregnancy outcomes are better for patients who have undergone surgery for TOF or TGA. However, pregnancy outcomes remain poor for patients with univentricularity after surgery.

In our study, there were 95 cases (88%) of pregnancy termination via surgery, with 75 of them due to cardiac intolerance. A previous study reported a CS rate of 44.8% for CCHD. The likely reason for the discrepancy is the different patient composition, as highly CCHD patients only accounted for 11.4% in that study. Previous studies have shown that significant haemodynamic fluctuations in pregnancy are associated with susceptibility to cardiovascular events^22,23^. Transvaginal delivery is highly susceptible to heart failure^24^. The American Heart Association’s stated that VD with adequate analgesia, supplemented by assisted labor, should be selected whenever possible, and CS should be selected according to obstetric indications^7^. Currently, there is no uniform clinical standard for the mode of delivery of patients with CCHD, which should be based on the patient’s cardiac condition^25^.

In addition to pregnancy risk, the offspring outcome of pregnant women with heart disease has also become the focus of current research in China and foreign countries. According to the National Birth Defects Prevention Network (NBDPN), birth defects occur in approximately 3–5% of newborns^26^. CHD is the most common birth defect, and its genetic mechanism remains to be fully understood. It is currently known that chromosomal abnormalities, copy number variations, and genetic defects occur in about 8–12%, 3–25% and 3–5% of patients with CHD, which may be inherited to their offspring. Therefore, it is recommended that women with CCHD undergo genetic testing before pregnancy. Studies have shown that 3–20% of patients with CHD can pass on the defect to their offspring, and such a risk of female patients is about twice that of male patients^27^. The proportion of newborns with birth defects was similar to that in the total population, but the proportion of newborns with CHD was significantly lower. However, this warrants further investigation. According to the ROPAC, the number of pregnant women with heart disease has increased significantly worldwide over the past five decades, and CCHD accounts for 20% of patients with CHD (66%), mostly with class III-IV cardiac function according to the modified World Health Organization (WHO) classification^4^. Such patients still have greater maternal and fetal risks during pregnancy, so close monitoring before, during and after pregnancy is required, and delivery plans should be formulated in advance to avoid as much as possible adverse outcomes such as maternal malignant arrhythmia, heart failure, fetal growth restriction and premature delivery^28^. To sum up, pregnancy is not recommended for CCHD patients with BNP > 100 pg/mL, no cardiac surgical correction and hypoxemia, and cardiovascular disease should be corrected first before pregnancy to obtain better maternal-fetal outcomes.

There are several limitations to this study. First, the patients were from a single cardiac center, and the cases span a long period during which surgical treatment levels have progressed. It is impossible to exclude data deviations caused by differences in surgical techniques at different centers and in different years. Second, due to the long case duration and changes in patient contact information, complete follow-up information was not obtained, limiting the exploration of long-term prognosis. A large number of prospective studies are still needed. Third, considering the small sample size, the use of propensity score matching may introduce confounding bias, and the significance of cardiac surgical correction needs to be confirmed by more prospective studies.

In conclusion, in this study we find the occurrence of adverse pregnancy outcomes is associated with BNP > 100 pg/mL, hypoxemia, NYHA cardiac function class ≥III, no cardiac surgical correction and elevated pulmonary arterial pressure, and BNP > 100 pg/mL, hypoxemia and no cardiac surgical correction are independent risk factors for adverse pregnancy outcomes. Cardiac surgical correction before pregnancy in CCHD patients is associated with reduced risk of deterioration of cardiac function, heart failure, hypoxemia, anemia, pulmonary arterial hypertension, premature delivery, postpartum hemorrhage, low birth weight and neonatal complications (infection, asphyxia) as well as the length of SICU stay.

## Supplementary information

Supplementary Information

## Data Availability

The databases for this study are available from the corresponding authors upon reasonable request.
