# Supplementary material for: Pregnancy outcomes among patients with complex congenital heart disease
Source: NPJ Cardiovasc Health. 2024 Sep 17;1:20. doi: 10.1038/s44325-024-00022-w (PMC12912378; doi:10.1038/s44325-024-00022-w)
Supplement: Supplementary file 1 — Supplementary Information [file 44325_2024_22_MOESM1_ESM.pdf]

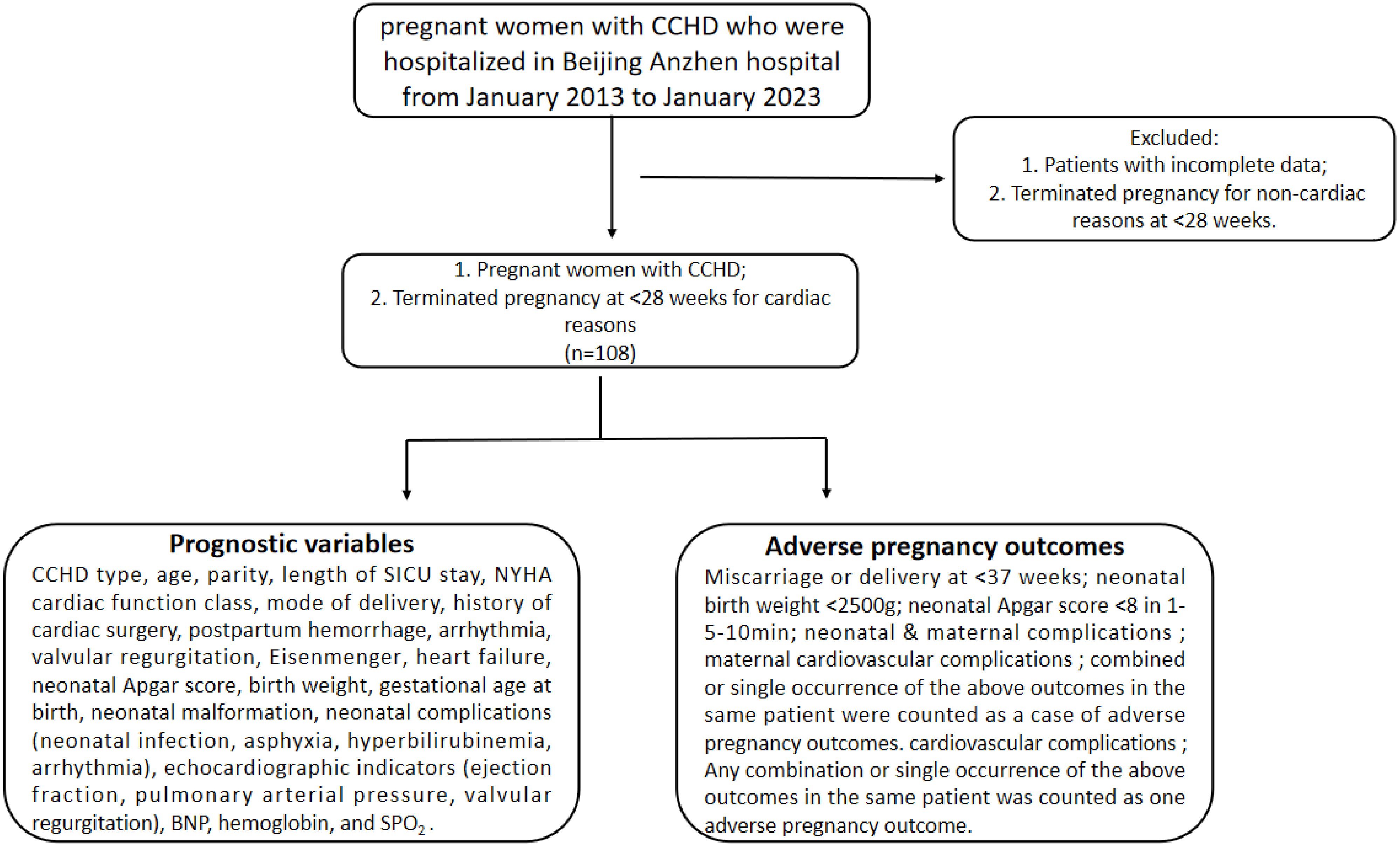

Supplementary Figure 1. Flowchart of the current study showing the recruitment of patients and variables analysed for included patients.
